# The Influence of MHC and Immunoglobulins A and E on Host Resistance to Gastrointestinal Nematodes in Sheep

**DOI:** 10.1155/2011/101848

**Published:** 2011-04-12

**Authors:** C. Y. Lee, K. A. Munyard, K. Gregg, J. D. Wetherall, M. J. Stear, D. M. Groth

**Affiliations:** ^1^Curtin Health Innovation Research Institute and Western Australian Biomedical Research Institute, Curtin University, Perth, WA 6845, Australia; ^2^Department of Animal Production and Public Health, School of Veterinary Medicine, University of Glasgow, Bearsden Road, Glasgow G61 1QH, UK

## Abstract

Gastrointestinal nematode parasites in farmed animals are of particular importance due to their effects on production. In Australia, it is estimated that the direct and indirect effects of parasite infestation cost the animal production industries hundreds of millions of dollars each year. The main factors considered by immunologists when studying gastrointestinal nematode infections are the effects the host's response has on the parasite, which immunological components are responsible for these effects, genetic factors involved in controlling immunological responses, and the interactions between these forming an interconnecting multilevel relationship. In this paper, we describe the roles of immunoglobulins, in particular IgA and IgE, and the major histocompatibility complex in resistance to gastrointestinal parasites in sheep. We also draw evidence from other animal models to support the involvement of these immune components. Finally, we examine how IgA and IgE exert their influence and how methods may be developed to manage susceptible animals.

## 1. Introduction

Gastrointestinal worm infestation is one of the major causes of reduced productivity in domestic sheep in tropical and temperate regions of the world. In common with other parasitic infections, there is a complex interaction between the host's innate and adaptive defence mechanisms and consequent adaptations by the parasite. An understanding of these interactions is essential for the development of sustainable strategies to minimise the impact of the parasite burden on the host. Analysis of the problem is made more difficult by the diversity of nematode species and strains that commonly infect sheep and the apparently variable manner in which sheep respond to these organisms. 

Inherited factors play an important role in determining susceptibility to nematode infections. For example, over the past two decades, the Rylington Merino Project has selected sheep for resistance to nematodes on the basis of annual worm egg counts [1, 2]. Relative to a control flock, the selected flock now has sufficient inherited resistance to nematodes that anthelminthic chemicals are not required during the lambing season. Selective breeding has been successful in other research flocks [1, 3, 4] and many commercial farms. Resistant animals can be identified by measuring faecal egg counts (FECs) over the first year of life. Selection for nematode resistance is widely practised in Australia and New Zealand but less common in the rest of the world. 

In Australia and New Zealand, the correlations between FEC and growth rate have been weak [5–7]. In contrast, in Europe, the correlations are strong [8–10] but have been shown to change over time. The differences may reflect the breed of sheep in the different regions, that is, Australian Merino, New Zealand Romney, Scottish Blackface, and Polish long wool sheep. Alternatively, the differences may be a consequence of the nematode community. In the two European FEC studies, egg counts were predominantly *Teladorsagia circumcincta *but in the Australian and New Zealand studies, *Haemonchus contortus *or *Trichostrongylus colubriformis* made a much greater contribution to egg counts. Alternatively, the differences between Europe and Australasia could reflect the different husbandry conditions; European sheep generally reach sale weights at an earlier age. IgA and IgE responses have been associated with reduced egg counts, but IgE responses have been shown to develop more slowly and are associated with pathology [11]. 

Many studies have implicated variation within the major histocompatibility complex (MHC) as a determinant of host resistance and/or sensitivity to gastrointestinal parasitism in several species [12]. In addition, mucosal humoral responses to parasites have been implicated in mechanisms that restrict parasite growth and mediate the expulsion of worms [13]. In this paper, the roles of the MHC and immunoglobulin synthesis, especially IgA and IgE, are discussed with particular emphasis on nematode infections in sheep.

## 2. Role of Adaptive Immunity in Gastrointestinal Parasitic Infestation

Parasitic gastroenteritis is caused by nematodes that include species from the genera *Trichostrongylus*, *Teladorsagia*, *Haemonchus, Nematodirusi, *and* Cooperia* [14]. Infections usually arise from ingestion of parasite larvae or eggs from pasture, and it is well established that the presence of parasite antigens in the host's gastrointestinal system triggers innate immune responses, in addition to humoral and cell-mediated adaptive responses, with recruitment of T cells along the gastrointestinal mucosa [15, 16]. During an initial infection, dendritic cells take up and process parasite molecules. The dendritic cells then migrate to the draining lymph nodes and activate T cells, although additional interactions between antigen presenting cells and T cells may occur close to the site of uptake. In the small intestine, soluble antigens (metabolic or excretory-secretory components) are absorbed by specialised microfold cells in the follicle-associated epithelium overlying the Peyer's patches either through phagocytosis or pinocytosis [17]. Antigens are transported from the intestinal lumen to the subepithelial dome, where the antigen-presenting cells interact with T cells. 

The importance of T lymphocytes, which regulate the host adaptive response against gastrointestinal parasites, has been demonstrated in several laboratory animal models, including *Trichinella spiralis*, *Heligmosomoides bakeri*, and *Strongyloides stercoralis* [12, 37, 38] and also in sheep infected with *Haemonchus contortus* [39]. However, it is also clear that adaptive immune responses to nematode parasites do not completely prevent subsequent infection, at least in most animals within a flock. 

The three major manifestations of resistance to nematodes are reduced numbers of adult nematodes, decreased size of adult nematodes, and increased numbers of inhibited larvae, compared to susceptible contemporaries. However, not all resistant animals manifest all the three primary indicators, and the three indicators do not develop at the same rate [40, 41]. Large worms tend to lay more eggs [42] and are generally more pathogenic [11]. Reduced egg counts, increased expulsion of parasites, altered growth rates in resistant hosts, increased numbers of eosinophils, mast cells, plasma cells, and lymphocytes as well as increased concentrations of antibody are common secondary indicators in most nematode infections of sheep. 

Much of the current knowledge concerning the mammalian immune response to parasites comes from studies on laboratory animals, particularly rodents. Experimental infections in rodents have provided valuable information for the analysis of immunological and genetic mechanisms that determine resistance to gastrointestinal nematode parasites [32, 43]. The demonstration that genetic factors influence resistance and susceptibility in mice allows the identification of genetic markers or genes that confer resistance [43]. Although the genes controlling resistance in different species are unlikely to be identical, many of the pathways are likely to be similar.

## 3. The Role of IgA in Nematode Resistance

In several host-parasite systems, parasite-specific IgA has been associated with resistance [44–48]. However, careful experimental design and interpretation are needed because IgA responses to nematode infection are correlated with IgE production, together with infiltration of eosinophils and mast cells and the subsequent degranulation of mast cells [49]. The mutual correlations could be a consequence of cytokines from Th2 cells, which recruit the relevant cells. Therefore, it is possible that increased IgA activity may be a marker of an increased mucosal immune response. IgA is not complement fixing and recently has been implicated in anti-inflammatory mechanisms [50]. Evidence for an active role is discussed below. 

In mice, the humoral immune response has been reported to exert a direct effector role against gastrointestinal nematode parasites. Immunity against murine *Trichuris muris* has been achieved through monoclonal IgA antibody infusion that resulted in the expulsion of the parasites from the gastrointestinal tract [51]. The immune mechanism was thought to be through antibody binding directly to parasite excretion/secretion antigens [51]. 

Smith et al. [52] were the first to report a relationship between IgA response and reduced worm length following infection with *T. circumcincta.* They examined the length of all nematodes, including larval stages, to identify inhibited larvae. They found an increase in lymphatic IgA and IgA-positive cells in the gastric lymph. Pooling data across age classes produced an extremely strong correlation between the increased IgA response and increased numbers of inhibited larvae. A large study in naturally infected sheep supported this finding by showing that lambs with higher peripheral IgA activity against fourth-stage larvae showed inhibition of a higher proportion of larvae [53]. 

More recent data have cast doubt on the role of IgA in nematode inhibition [54]. Sheep were trickle-infected, then, challenged with 50,000 *T. circumcincta*. Parasite development ceased approximately five days after challenge and preceded the peak of IgA activity in the gastric lymph on day 9. The IgA response was apparently too slow to play a direct role in the inhibition of larval development. However, more research is necessary before firm conclusions can be made. The relationship between IgA levels in the gastric lymph and IgA levels at the site of infection in the abomasal mucosa is unknown. In addition, there is density-dependent inhibition of larval development [55]. The mechanism of density-dependent inhibition may differ from that of immune-mediated inhibition, and the inhibition observed in this experiment may not have been immune mediated. 

In contrast to the uncertain relationship between IgA level and numbers of inhibited larvae, the parasite-specific IgA response is consistently correlated with a reduction in adult worm length in infected animals. In Scottish Blackface sheep matched for age, sex, breed, farm of origin, and parasite exposure history, Stear et al. [49] observed considerable variation in the number of IgA-positive plasma cells and the activity of parasite-specific IgA in the abomasal mucosa. There was a negative correlation between IgA and worm length, which was stronger for mucosal IgA than for serum IgA. The correlations observed were also stronger against fourth-stage larvae (L4) than against third-stage larvae (L3). Recently, Henderson and Stear [56] showed a direct correlation between mucosal IgA and plasma IgA levels of 0.66. The negative correlation observed between parasite-specific IgA levels and worm length was likely to have been a direct effect of IgA on the parasite, rather than a change in the quantity of antibody produced in response to changes in worm number [49]. Similar correlations have been observed in Santa Ines, Suffolk, and Ile de France lambs infected by *H. contortus*, Scottish Blackface lambs infected by *H. contortus*, and Churra lambs infected with *T. circumcincta *[57–59]. In addition, Scottish Blackface lambs that were naturally infected with *T. circumcincta* have shown a similar relationship [53, 60]. 

Stear et al. [49] estimated that approximately 38% of nematode parasite worm length variation could be accounted for by mucosal IgA activity directed against L4 worms, a value considerably less than the over 90% estimated by Smith et al. [52]. However, the high value reported by Smith et al. may have been an artefact created by pooling data from sheep of different ages. The level of variation in nematode parasite worm length due to L4 parasite-specific IgA activity has been independently estimated as ~38% in Churra sheep [59], with similar estimates reported by Sinski et al. [61], Strain and Stear [57], Strain et al. [60], Stear et al. [53], Amarante et al. [58], and Henderson and Stear [56]. 

In addition to the effects of IgA, two other factors influence the size of adult nematodes: IgA specificity and worm density dependence. Variance analysis in sheep intentionally infected with *T. circumcincta* [53] indicated that these three components accounted for most of the variation in adult female worm length. This conclusion is consistent with the hypothesis that, in this host-parasite system, IgA is the major host mechanism influencing parasite growth and fecundity. In *Strongyloides ratti*, the density-dependent response is abolished in immunosuppressed rats [62], which suggests that density dependence is mediated through the immune system in at least some host-parasite systems.

There are several methods by which IgA could influence nematode growth. Parasitic nematodes release a variety of proteases that partially predigest proteins and may also break down antibodies and other mediators of host resistance. Antibodies against these enzymes or other molecules could inhibit enzyme activity and feeding by the parasite [63–67]. This appears to be a mechanism underlying the success of vaccination against H-Gal-GP (a galactose-containing glycoprotein complex purified from intestinal membranes of adult *H. contortus* worms) from *H. contortus *[68, 69]. Alternatively, IgA could interact with eosinophils to control nematode growth and fecundity (see below). 

There does not appear to be a consistent association between IgA activity and the number of adult *T. circumcincta *[49]. There is also no consistent association with the number of *H. contortus* [70–72]. The absence of a relationship suggests that IgA activity does not determine worm numbers.

Hertzberg et al. [73] trickle infected White Alpine lambs with *Ostertagia leptospicularis * and showed that there was a gradual increase in serum IgA levels during infection. As expected from other species, IgA has a short half-life and IgA activity declined rapidly after anthelminthic treatment. When subsequently challenged with 100,000 infective L3 parasites, the serum IgA level rose rapidly but was observed to decrease earlier than either IgG1 or IgG2.

## 4. IgA and Eosinophilia

Variation in the number of mast cells, globule leucocytes, eosinophils, and IgA plasma cells has been observed in sheep that were infected with nematodes [49, 58]. Globule leucocytes are derived from subepithelial mast cells [74, 75]. Stear et al. [49] found that sheep with more mast cells had higher abomasal concentrations of globule leucocytes, eosinophils, IgA plasma cells, and more larval antigen-specific IgA antibody. Henderson and Stear [56] measured the level of IgA and eosinophil numbers in Scottish Blackface lambs over a period of 60 days after challenge and observed that both variables had similar response kinetics. IgA and eosinophil activity peaked at 8–10 days after infection and declined subsequently. Stear et al. [49] measured eosinophil numbers at the end of the experiment during necropsy of the animals while Henderson and Stear [56] measured mucosal eosinophilia over a 60-day period. A similar study using Caribbean hair sheep and wool sheep [19] found that the hair breed had higher serum levels of IgA and IgE in uninfected sheep, and that there were significant differences in IgA, IgE, and tissue eosinophils levels between the two sheep breeds which was negatively correlated with worm counts. IgA levels accounted for 38% and eosinophil numbers 40% of the variation in worm length, respectively. In correlation studies that analysed the two variables together, the combination accounted for 53% of worm length variation. Therefore, it appears that IgA and eosinophilia have a combined or synergistic effect on worm length [56]. Eosinophils have been shown to express receptors for IgA [76, 77], which can be activated by binding of parasite antigen/IgA to IgA cell surface receptors [78]. Therefore, IgA could help target eosinophils to nematodes. Interestingly, eosinophils in mice lack receptors for IgA [76], and this could explain the relative ineffectiveness of eosinophils in some murine models [79, 80].

## 5. The Role of IgE in Nematode Resistance

Increased numbers of mast cells is a hallmark of many nematode infections, and they have been implicated in the control of worm numbers in some but not all infections. For example, mast cells appear crucial for the control of *Trichinella spiralis *but not for* Trichuris muris* or *Nippostrongylus brasiliensis *[81]. Sheep that are resistant to *T. circumcincta* have increased numbers of mast cells or globule leucocytes compared to more susceptible contemporaries [49]. Similarly, mast cells are important for resistance to *H. contortus* [82, 83].

As binding of parasite molecules by cell-surface IgE is the major trigger for mast cell degranulation, IgE is implicated by default in resistance to nematode infection. An association between high plasma IgE activity against a high-molecular-weight allergen and low egg counts was reported in 20 lambs selected from a group of 72 naturally infected crossbred sheep [84]. A study using lymphatic cannulation to allow continuous assessment of the migrating immune cells from the intestinal mucosa and mesenteric lymph nodes showed differential changes in the expression of IL-5 in the afferent intestinal lymph in two lines of sheep selected for susceptibility or resistance to *T. colubriformis* [85]. Furthermore, in a parallel study by the same group, the resistant line had higher IgE in lymph than the susceptible line [86]. Naturally infected Texel lambs with high IgE activity against recombinant tropomyosin from *T. circumcincta* also had lower egg counts than lambs with lower IgE responses [87]. An independent study from New Zealand also showed an association between increased IgE activity against an aspartyl protease inhibitor from *T. colubriformis* and reduced egg counts [88].

## 6. Genetic Factors in Gastrointestinal Parasite Immunity

Quantitative genetic analysis in sheep and cattle has clearly shown that resistance to nematode infection is under genetic control [2, 89–93]. The heritability of a single egg count varies among populations but is usually between 0.2 and 0.4 in animals that have been previously exposed to infection [94]. This is similar to the heritability of milk production in dairy cattle or growth rate in beef cattle and indicates the feasibility of selective breeding [95]. Quantitative trait loci (QTLs) for resistance to the intestinal nematode *Heligmosomoides polygyrus* were located on mouse chromosomes 1, 2, 8, 13, 17, and 19 by Iraqi et al. [32]. Interestingly, one chromosomal region identified by these researchers was the MHC located on mouse chromosome 17. Their observations were confirmed independently by Behnke et al. [33] who found associations between eight immunological traits (FEC at weeks 2, 4, and 6, mucosal mast cell protease 1, granuloma score, IgG1 against L5, and IgG1, and IgE to L4) and QTLs on chromosome 1 and 17 associated with resistance to the *H. polygyrus* infection. More specifically, the MHC genes, most notably, the class II and TNF regions were significantly associated with gastrointestinal parasite infection. 

Davies et al. [29] provided evidence of QTLs located on sheep chromosomes 2, 3, 14, and 20 conferring resistance to infection with *T. circumcincta* in Scottish Blackface sheep. Analysis of chromosome 20 showed that the MHC region had a statistically significant association with gastrointestinal nematode parasite resistance. QTLs associated with specific IgA activity against nematode parasites were also located on chromosomes 3 and 20. Alleles of the *DRB1* in the MHC class II region have been associated with nematode resistance in several different breeds of sheep [23–25, 96] and cattle [90, 97, 98]. However, in contrast, Beh et al. [99] found no significant linkage of the MHC in sheep resistance to *Trichostrongylus colubriformis*. Unfortunately, their study used only a single marker to represent the MHC region and chromosome 20 in their whole-genome linkage analysis. Beh et al. [99] also applied an additional two markers to a single-point ANOVA and confirmed no linkage to the MHC region. In another linkage study, no significant QTL was found on chromosome 20, for resistance to parasitic nematode infection in sheep [100]. In this study, only four markers were used to represent chromosome 20, of which only two mapped to the MHC region [100]. Recently, a more extensive whole-genome QTL analysis for resistance to *H. contortus* showed, in one family, weak linkage between egg counts and the *Ovar-DYA* region in the MHC class IIb region [101], consistent with a previous report that associated this region with resistance to *T. circumcincta *[26].

## 7. The Influence of the MHC on Antibody Production

The role of MHC in controlling IgA concentrations is supported by several human studies, especially on IgA and combined variable immunodeficiency (CVID). One of the first studies that identified an association between IgA deficiency and the MHC region was by Wilton et al. [102], who found an association between MHC class III genes and IgA deficiency. An increase in frequency of certain HLA haplotypes was observed in deficient patients [102, 103]. A number of studies have since focused on the *HLA-A1-B8-DR3* haplotype to locate the IgA deficiency locus [104, 105]. An investigation of the *HLA-DR3*-extended haplotype showed that in the Sardinian population, where a lower prevalence of IgA deficiency exists, the *HLA-DR3-B18* haplotype is more common than the *HLA-DR3-B8* haplotype, suggesting that the IgA deficiency susceptibility gene is located in the more common Northern European DR3-B8 haplotypes [106]. The investigation of features common to the different haplotypes was used to establish the region associated with IgA deficiency, and thus far several different studies have placed the susceptibility locus between the class III region [103, 105, 107, 108] and the class II region [109–111]. 

Polymorphisms in *MSH5* have also been shown to be associated with CVID and IgA deficiency in a mouse model and through statistical analysis of human populations [112]. This gene, located within the MHC class III region, is involved in DNA mismatch repair as well as in resolving Holliday junctions that form between homologous DNA strands during meiosis [113, 114]. However, Guikema et al. [115] observed a large variety of splice variants of MSH5 mRNA (all of which are unlikely to be stable) and suggested that *MSH5* was nonfunctional and therefore probably does not participate in Ig class switching. Recently, it has been shown that haplotypes of *MSH5* are associated with IgA deficiency [116, 117] but are not likely to be the causative mutations [117].

## 8. Mechanisms Underlying the MHC Association with Nematode Resistance

Genetic variation in the mouse MHC has long been associated with resistance to nematode infection [118] and with the specificity of antibody responses [119]. It has been reported that the helminth *Nippostrongylus brasiliensis* may possibly be able to suppress MHC class II molecule expression as an evasive mechanism [120]. Likewise, for sheep, it has been shown that the parasite *T. colubriformis* seems to be capable of downregulating several immune genes, particularly *DRB1* and *DRA*, in afferent lymph migratory cells [121]. In the mouse model infected with *Strongyloides venezuelensis*, class II ^−/−^ animals were more susceptible to infection (based on increase in FEC and elimination of worms) than wild-type and class I ^−/−^ mice [31]. In addition, parasite-specific IgM, IgA, and IgG were also significantly reduced in class II ^−/−^ mice. This study concluded that class II MHC expression was essential to induce a Th2 response against *S. venezuelensis* infection and class I expression was not [31]. Interestingly and somewhat contradictory to the findings discussed above [121], it has been shown that mice strains that lack *I-E*, a homologue of *DRB1*, in their MHC class II region are more resistant [122].

In a comparative study using bovine cDNA microarray analysis of duodenum tissue from an outbred population of resistant and susceptible lambs (which had been subjected to two natural challenges with a range of gastrointestinal parasites), increased expression was observed in a range of genes [18]. Upregulated genes included *DQB1*, *DRA*, and *DQA1* from the MHC class II region [18]. This observation highlights key differences between resistant and susceptible animals in the early immune response to gastrointestinal nematodes. In a separate microarray study, differences were observed in gene expression profiles of hair and wool sheep that had been infected with *H. contortus* [19]. Elevated expression of the MHC class II DM *β*-chain precursor gene was observed in lymph node tissue of the wool breed. However, no significant change in the expression of this or any other MHC-related gene was observed in abomasal tissue [19]. In another study, using transcriptional profiling of duodenum tissue samples from resistant and susceptible sheep [20], up-regulation of MHC class II genes *Ovar DQA1*, *Ovar DQB1*, and *Ovar DRA* was observed in resistant animals. Subsequent RT-PCR analysis of *Ovar DQA1 *showed an average 8.4-fold greater expression in resistant animals than in susceptible animals. Further analysis using GO terms highlighted the significant association between genes highly expressed in resistant animals with terms such as MHC class II activity and exogenous antigen processing and presentation [20]. Furthermore, the frequency of *Ovar DQA1* haplotypes differed between animals from the resistant and susceptible selection lines, with an increase in Ovar DQA1*Null in susceptible animals from both Perendale and Romney sheep lines. In Perendale sheep, the frequency of Ovar DQA1*0101 and DQA1*0402 alleles was increased in resistant animals and Ovar DQA1*0103 increased in the susceptible line. However, these observations seemingly contradict earlier findings by the same group, in which no increase was observed in the expression of either MHC class II genes nor any association was found with antigen presentation or processing [123]. Interestingly, a significant increase in expression of a MHC class I gene (*HLA-A* orthologue) in resistant animals was also observed, indicating possible crosstalk between the different responses. Recently, Forrest and colleagues [21] conversely demonstrated no evidence of an interbreed effect of the Ovar-DQA1*Null allele on total faecal egg counts. However, the Ovar-DQA1*Null appeared to have a significant effect when the analysis was performed within breeds [21].

In a statistical examination of the relationship between MHC polymorphism and parasitological traits in Scottish Blackface sheep, the resistant allele G2 at the DRB1 locus was significantly associated with decreased egg counts and decreased numbers of adult *T. circumcincta* [96]. However, no apparent correlation was observed with adult female parasite length. Hence, the mechanism by which the MHC influences egg counts may operate through the control of worm number and not by controlling nematode fecundity. There are several possible mechanisms but possibly specific class II molecules direct responses to specific peptides, and these responses may play a direct role in protection. 

Another possibility is that the observed associations in livestock are a consequence of heterozygote advantage [96]. Heterozygote advantage has complex effects on the power of statistical analyses to detect specific allele effects [27]. As the frequency of an allele increases in a population, an increasing proportion of homozygous sheep will be present and thus the average effect of the specific allele will decline. Also, an allele that is very rare in a population will be present in too few animals to show a significant effect. Conversely, when the allele is very common in the population, its average effect is quite small making its contribution to reduced egg counts difficult to detect. Consequently, only alleles within a narrow frequency range will show effects on parasite resistance. Interestingly, the allele most strongly associated with resistance in Scottish Blackface sheep fell within the narrow detection window, and the most common allele was also associated with the most susceptible animals as predicted by heterozygote advantage. There was also more direct evidence: Heterozygous sheep had lower egg counts following natural *T. circumcincta *infection [96]. 

Heterozygote advantage is a particularly appealing mechanism for explaining the IgE response to parasites. The specificity of IgE responses is relatively unimportant for mast cell degranulation if the target molecule is soluble and large enough to promote cross-linking of IgE receptors. Therefore, a heterozygote advantage that leads to increased IgE concentrations is more supported than a model of determinant selection (i.e., a direct role of the allele in determining levels of IgE).

Charbonnel and Pemberton [124] examined both MHC and neutral loci in free-living Soay sheep that were infected by *T. circumcincta* in St Kilda (Scotland). Over eight years, lower levels of temporal genetic differentiation were observed at MHC loci compared with neutral loci, consistent with balancing selection activity at the MHC loci [124]. These observations confirmed earlier work by Paterson [125] but have not been supported by subsequent research [126]. Significant studies showing positive associations between genes within the MHC and gastrointestinal parasites are summarised in Table 1.

## 9. Conclusions

There is no single mechanism of nematode resistance in sheep. Resistance to gastrointestinal nematodes involves the control of worm growth as well as worm numbers. The negative correlation between parasite-specific IgA levels and worm length has been well established by many research groups in different breeds of sheep infected by different gastrointestinal parasites. The control of worm numbers involves mast cells in some but not all host-nematode systems. There is a genetic component to nematode resistance, and the MHC is one of the most important components of genetic resistance. QTL analyses have shown a link between the MHC region and FEC in mouse models, as well as in sheep and cattle. The influence of the class II region on parasite resistance has been shown in experimental models as well as by microarray analysis. 

Despite the large number of studies that confirmed these relationships, there are other studies in which contradictory results reject these hypotheses. However, correlation studies may generate a complex heterogeneity of results because of the large variety of gastrointestinal nematode parasites and differences in environmental conditions, nutritional status of animals, and geographical locations. Another complication is that the relationship between gene expression from the MHC region, IgA activity, and their effects on parasites is often considered individually rather than as interconnecting multilevel interactions.

## Figures and Tables

**Table 1:** Summary of studies that have implicated the MHC in resistance to gastointestinal parasites.

Species	Parasite species	Method	MHC association	Reference
Sheep (*Ovis aries*)	mixed	Microarray	*DQB1, DRA, DQA1*	[18]
	*H. contortus*	Microarray	*DMB*	[19]
	mixed	Microarray	DQA1*Null, DQB1, DRA	[20]
	mixed	PCR analysis	DQA1*Null	[21]
	mixed	PCR/sequencing	DQA1*0101, DQA1*0402	[20]
	mixed	PCR/sequencing	*DRB1*	[22, 23]
	mixed	PCR	*DRB microsatellite*	[23]
	*Teladorsagia circumcincta*	PCR/sequencing	*DRB1*	[24–27]
	*H. contortus*	PCR/sequencing	*DRB1, OMHC1*	[28]
	*Teladorsagia circumcincta*	Linkage	Class IIb region	[29]
				
Sheep (*Ovis canadensis*)	*n/a*	Population analysis PCR/sequencing	*DRB1*	[30]
				
Mouse (*Mus musculus*)	*S. venezuelensis*	Knock out	Class II	[31]
	*H. polygyrus*	Linkage	Class II region	[32, 33]
				
Striped mouse (*Rhabdomys pumilio*)	mixed	PCR/sequencing	*DRB*	[34]
Yellow necked mouse (*Apodemus flavicollis*)	mixed	PCR/sequencing	*DRB*	[35]
Gray mouse lemur (*Microcebusmurinus*)	mixed	PCR/sequencing	*DRB*	[36]
